# Translating Behavioral Interventions: It Is More Than Just Language

**DOI:** 10.1093/geroni/igaa057.2157

**Published:** 2020-12-16

**Authors:** Katherine Marx, Laura Gitlin

## Abstract

In the United States, over 5 million people are living with Alzheimer’s disease or a related dementia. Providing care are an estimated 16 million unpaid caregivers and millions of paid caregivers. Neuro-psychologic symptoms (NPS) such as agitation, aggression, depression, rejection of care, and apathy are almost universal in persons living with dementia (PLwD). Caring for NPS often leads to poor physical, mental and financial outcomes. There have been hundreds of non-pharmacologic interventions tested and found efficacious to help caregivers with NPS and daily care challenges. However, very few of these interventions have been widely adopted in different languages and settings. One promising intervention used in various countries is the Tailored Activity Program (TAP). TAP, delivered by occupational therapists, customizes activities to PLwD’s current capabilities and prior roles and interests and instructs caregivers in their use. This session will examine TAP’s reach and how it has been translated and adapted. First, Ms. Sokha Koeuth will present modifications needed to the program to facilitate widespread dissemination including placing training in the program online and virtual. The next two presentations will discuss adaptations to TAP in different countries and cultures; Dr. Marcia Novielli will present TAP-Brazil, and Dr. Jean Gajardo Jauregui will present TAP-Chile. Finally, Dr. Katherine Marx will examine the adaptations needed to place TAP into a long-term care setting with both family and paid caregivers. These papers highlight the cross-cultural adaptations that need to be considered in taking a program from research to different real world clinical and community-based settings. Behavioral Interventions for Older Adults Interest Group Sponsored Symposium.

